# **Circumferent dissection of the ascending aorta resulting in the occlusion of supra-aortic vessels repaired using the frozen elephant trunk technique - a case report**

**DOI:** 10.1186/s13019-024-02765-0

**Published:** 2024-05-22

**Authors:** Frieda-Maria Kainz, Kathrin Freystaetter, Felix Nagel, Dominik Wiedemann, Bruno K. Podesser

**Affiliations:** 1 Karl Landsteiner University of Health Sciences, Dr. Karl-Dorrek-Straße 30, 3500 Krems, Austria; 2 Division of Cardiosurgery, University Hospital St. Pölten, Dunant-Platz 1, 3100 St. Pölten, Austria

**Keywords:** Type a aortic dissection, Frozen elephant trunk, Invaginated intimal mass

## Abstract

**Background:**

Our patient presented with acute back pain and dyspnea, without neurological symptoms. The computed tomography (CT) scan showed a circumferent rupture of the ascending aortic intima which was invaginated in the arch and descending aorta.

**Case presentation:**

A 54-year-old male patient was diagnosed with a Type A aortic dissection. He was immediately transferred to our operation room (OR) from the emergency department of a peripheral hospital. He presented with a circumferential dissection of the ascending aorta originating just distal to the coronary ostia, with the invaginated intimal mass extending through the arch down the descending aorta. In mild hypothermia, the intimal mass was safely extracted and a frozen elephant trunk (FET) procedure was performed.

**Conclusions:**

Despite the extensive dissection affecting the ascending aorta and aortic arch, resulting in partially occluded supra-aortic vessels by an intimal mass, the patient remained without neurological symptoms in the pre- and post-operative period and remains well one year post surgery.

## Background

Type A aortic dissection has high morbidity and mortality rates and presents an indication for emergency surgery [1]. The symptoms and signs are diverse [2]. In this case report we present a case of Stanford Type A aortic dissection with a circumferential rupture of the intima at the level of the sinotubular junction, resulting in the invagination of the intima extending into the aortic arch and descending aorta, occluding supra-aortic vessels. Notably, our patient presented awake without neurological symptoms.

## Case presentation

We report the case of a 54-year-old male patient, presenting to the local emergency department with acute chest pain and dyspnea, haemodynamically stable with a significant blood pressure difference on the upper extremities, neurologically fully intact. His medical history included an enlarged aortic root (52 mm) under surveillance, hypertension, a bilateral pulmonary embolism in 2014. An elevated D-Dimer (48700ng/mL) was the only abnormality in the initial laboratory results.

**Fig. 1:**
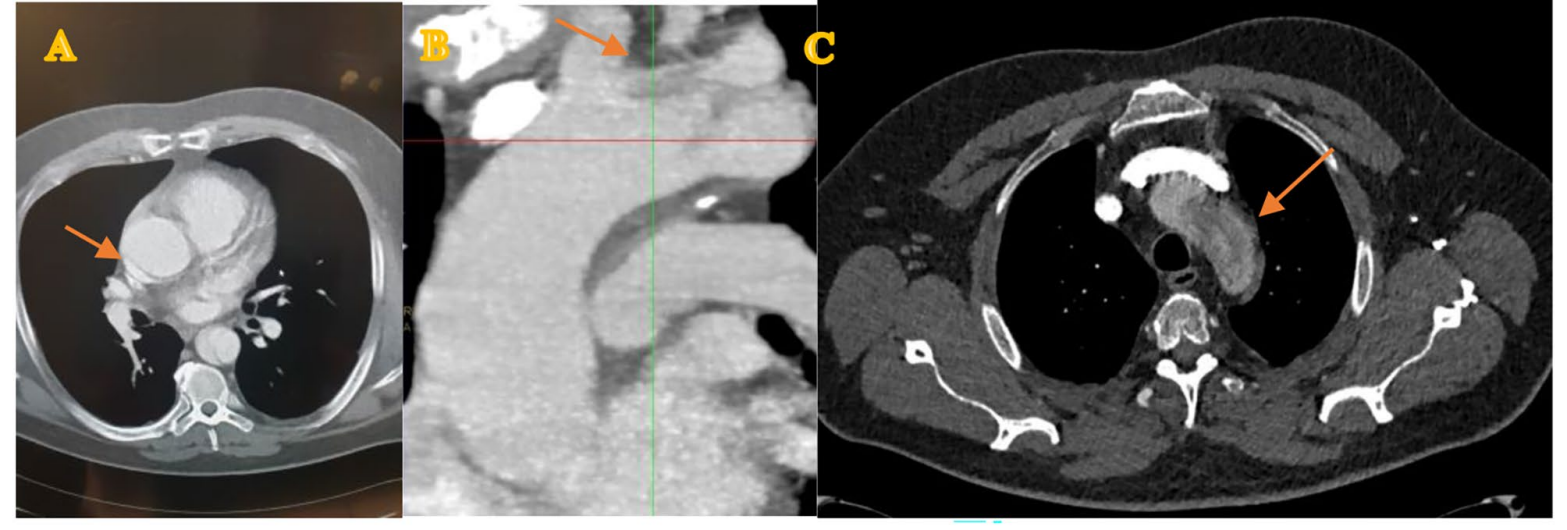
preoperative CT scan, A: small intima flap, B: intima flap in the aortic arch, C: dissection membrane in the aortic arch

Computed tomography (CT) showed an acute aortic dissection, the dissection membrane extending from the ascending aorta just distal to the coronary ostia down to the iliac vessels with multiple re-entries in the descending aorta at the level of sixth and eight intercostal space and the renal branches. The supra-aortic branches were not dissected, but occluded by an intimal flap (Fig. 1).

**Fig. 2:**
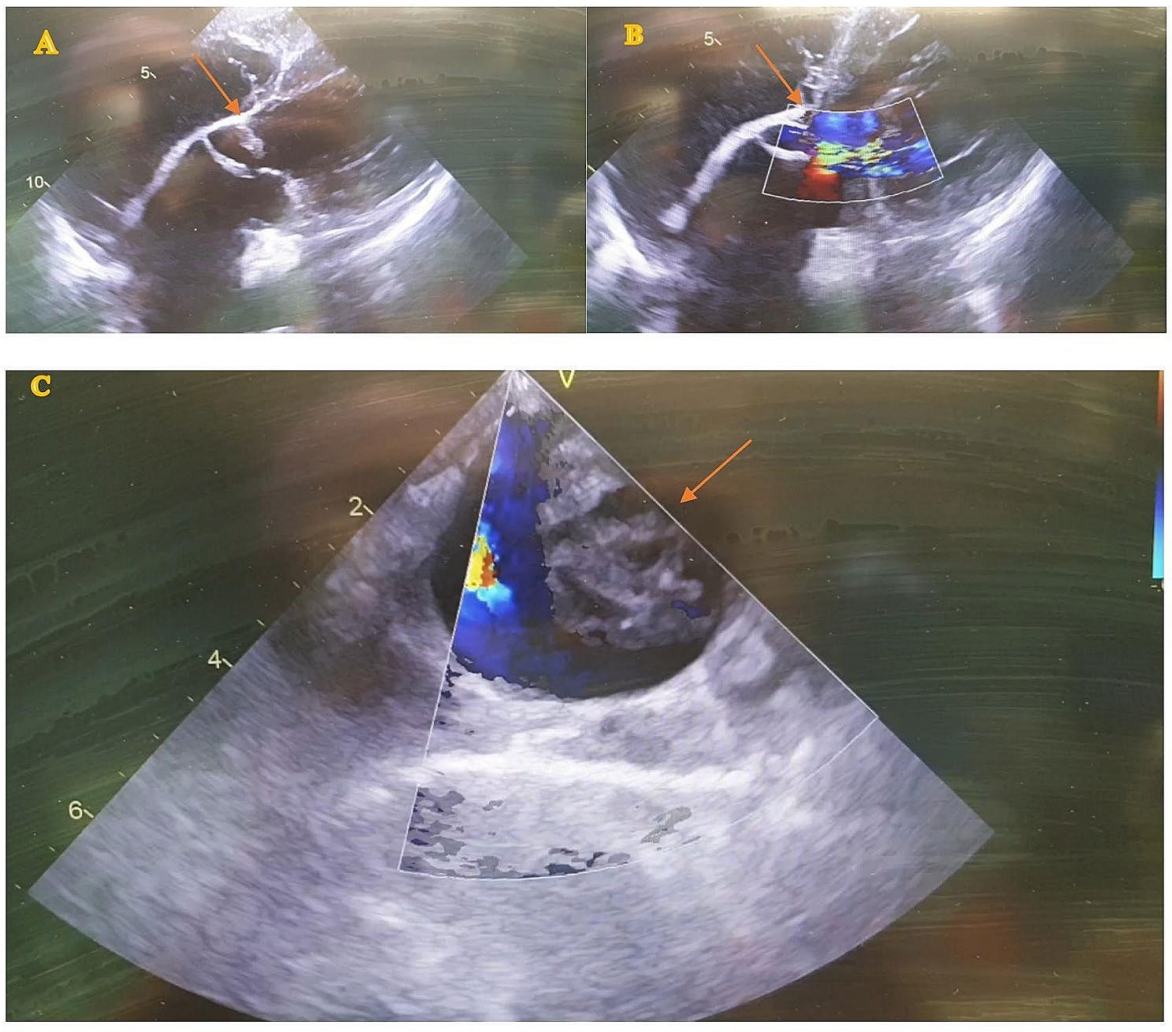
Intraoperative TEE depicting the intima flap in the ascending aorta, A: with, B: without color, C: descending aorta with flow and intima mass

Transoesophageal echocardiography (TEE) showed moderate to severe aortic valve regurgitation, a hypertrophic left ventricle with an ejection fraction of 40–50%, a flap in the ascending aorta as well as a mass extending from the ascending into the descending aorta (Fig. 2).

**Fig. 3:**
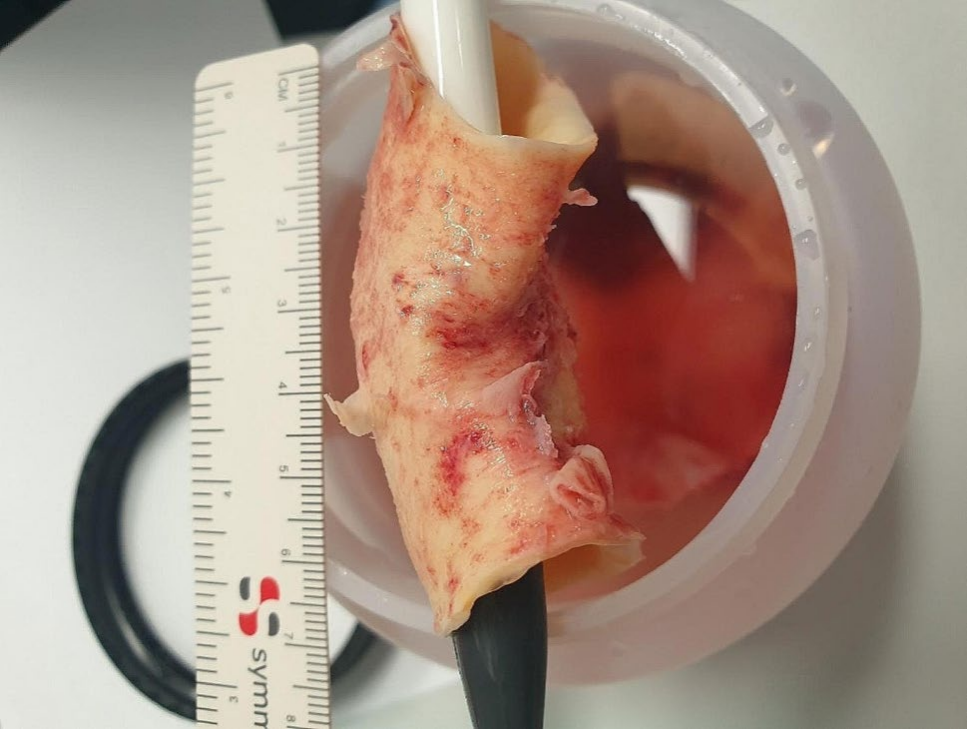
invaginated resected intimal mass

The patient was referred to our team for emergency surgery and transferred to our operating room. Following median sternotomy, the pericardium was opened and a bloody pericardial effusion was drained. Cardiopulmonary bypass (CPB) was established via the right subclavian artery and the right atrial appendage, retrograde cold blood cardioplegia was delivered via the coronary sinus, and the patient was cooled to 28 degrees Celsius, measured through the urin temperature catheter. The brachiocephalic trunk was cross-clamped and unilateral antegrade cerebral perfusion was initiated. Incision of the ascending aorta revealed only a small remainder of intima at the level of the sinotubular junction, no intima was visible in the ascending aorta. On inspection of the aortic arch and supra-aortic vessels no true lumen was visible. An intimal mass (Fig. 3) was invaginated into the distal aortic arch; this was retracted and carefully resected, revealing the supra-aortic branches.

It was now possible to insert the second cerebral perfusion catheter into the left carotid artery, commencing bilateral cerebral perfusion. The ascending aorta was resected, the left subclavian artery remained in situ due to its distal location. The distal aortic anastomosis was secured using felt strips in a sandwich technique with a 4/0 Prolene, a 28/30/12/8/10 × 100 mm Thoraflex frozen elephant trunk (FET) prosthesis was selected and secured with a 3/0 Prolene running suture. CPB was continued via the FET prosthesis, the patient warmed up to 34 degrees Celsius. Subsequently, the left carotid artery and brachiocephalic trunk were anastomosed to the prosthesis with 5/0 Prolene. Finally, the proximal aortic anastomosis was made at the level of the sinotubular junction, again using a felt strip sandwich technique. The final intraoperative TEE showed a competent aortic valve and good biventricular function.

**Fig. 4:**
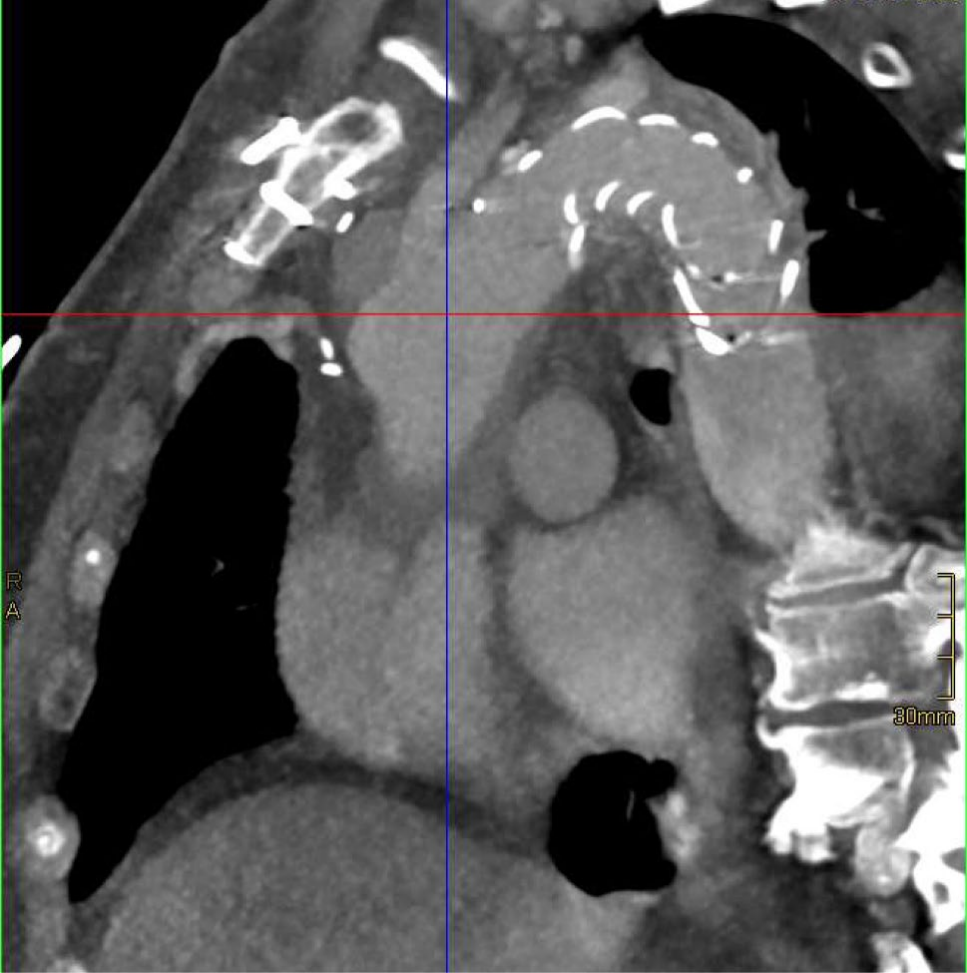
postop CT scan after one year

Postoperatively, the patient was transferred to the Intensive Care Unit (ICU), weaned and extubated on the second postoperative day (POD) and transferred to the ward on the sixth POD. Echocardiography and CT prior to discharge showed a regular postoperative result; a competent aortic valve, preserved left ventricular ejection fraction, a minimal pericardial efffusion, no signs of anastomotic dehiscence. The patient remained neurologically intact and was discharged from hospital on the 23rd POD. He remains well one year after surgery; a follow-up CT (Fig. 4) showing good postoperative results.

## Discussion

In 1962, Hufnagel and Conrad reported the first case of intimo-intimal intussusception in dissecting aneurysms. Since then, fewer than 80 cases have been documented in type A dissections. Roberts et al. reviewed 35 cases and Yamashita et all found 44 cases by April 2020. Complete or near-complete circumferential dissection in ty A poses significant risk due to mobile cylinder-shaped flaps. These flaps, categorized by Sanders et al., can intussuscept antegrade into the aortic arch or retrograde in the left ventricle, potentially leading to severe complications. Diagnosis often involves echocardiography and CT, with TEE being particularly value for real-time visualization and assesment [3]. 

The Frozen Elephant Trunk (FET) technique has demonstrated favorable outcomes in acute Type A aortic dissection, with notable complications including spinal cord injury, stroke, and acute kidney injury requiring dialysis [4]. 

There is ongoing debate regarding the routine use of FET, but recent evidence suggests it is associated with reduced reinterventions and improved long-term outcomes. By resecting intimal tears beyond the ascending aorta, the FET procedure can exclude distal re-entries using hybrid prostheses, re-expand the true lumen, and prevent complications such as degeneration, aneurysmal changes, or further dissection. FET may also prevent further dilatation of the descending aorta by covering secondary entries and obliterating the false lumen, ultimately leading to better outcomes. Additionally, FET facilitates secure distal anastomosis and reduces the risk of rupture or bleeding, even in emergency surgeries [5]. Notably, surgeons need not be proficient in endovascular stent delivery techniques, as the prosthesis can be safely inserted into the downstream aorta under direct view during circulatory arrest [6]. 

However, careful patient selection is crucial, considering clinical condition, symptoms, age, and comorbidities, along with the expertise of the surgical team, as prolonged operation time and increased complications may occur [7]. Long-term follow-up is necessary to refine the optimal approach for managing these complex cases.

## Conclusions

To the best of our knowledge this is the first case report of an invaginated intimal mass originating from the ascending aorta, obstructing supra-aortic vessels and extending into the descending aorta. The FET procedure provided a suitable solution for this ascending aortic dissection and destroyed aortic arch, showing favorable early results, positive remodeling of the aorta and a decreased need of reinterventions. Our patient remains alive and well with a favorable neurological outcome at 12 months.

## Data Availability

Data sharing is not applicable to this article as no datasets were generated or analyzed during the current study.

## Abbreviations

CT: computed tomography
CPB: cardiopulmonary bypass
FET: frozen elephant trunk
ICU: intensive care unit
INVOS: in-vivo optical spectroscopy
OR: operating room
POD: postoperative day
TEE: transoesophageal echocardiography
